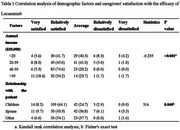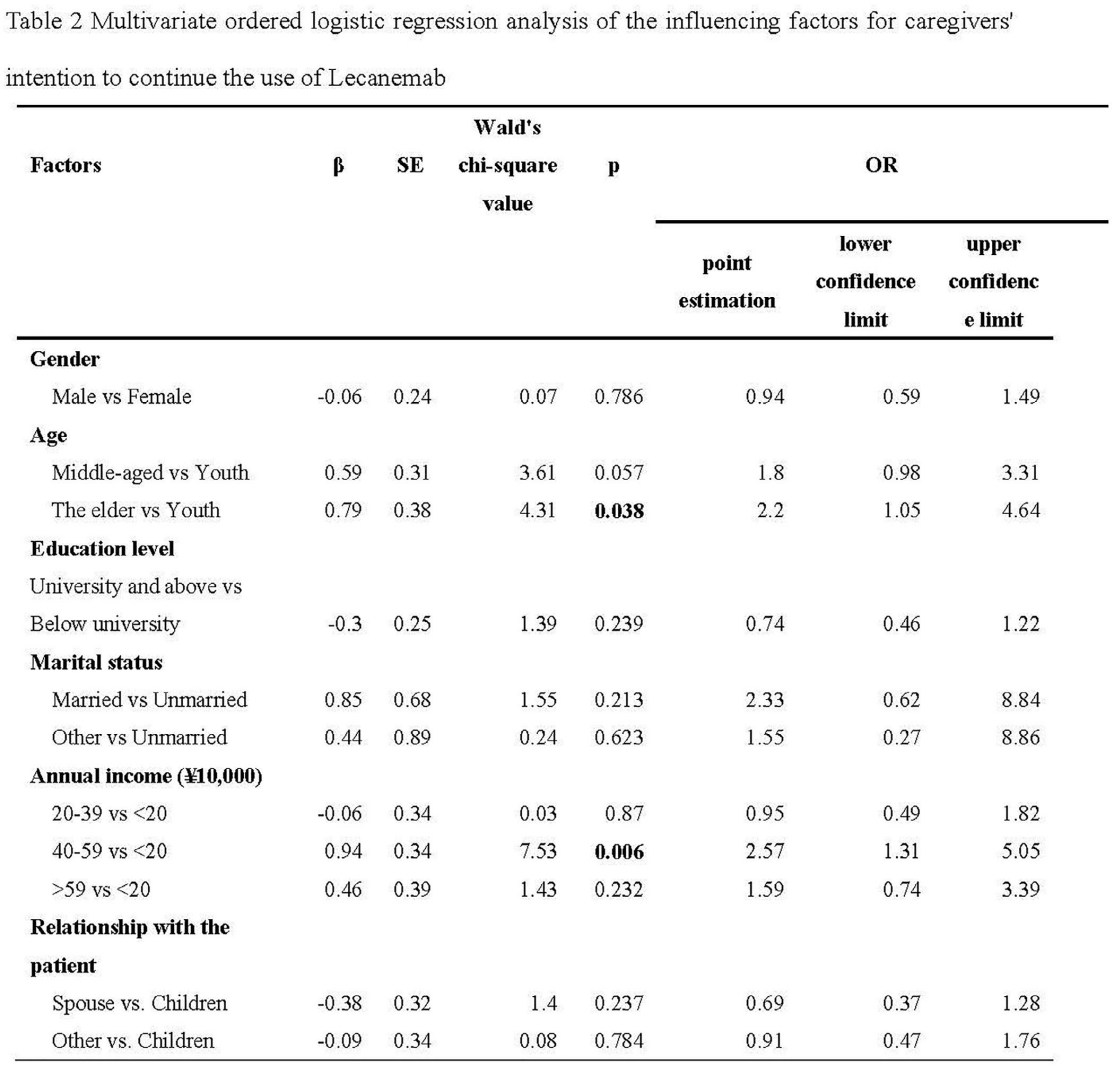# Caregivers' attitudes regarding the application of Lecanemab for Alzheimer's disease: a cross‐sectional survey in China

**DOI:** 10.1002/alz70858_099220

**Published:** 2025-12-25

**Authors:** Shuai Liu, Shiyu Fan, Jinghuan Gan, Wang Liao, Qin Chen, Xia Li, Jiewen Zhang, Xiaochun Chen, Yong Ji

**Affiliations:** ^1^ Department of Neurology, Tianjin Huanhu Hospital, Tianjin Key Laboratory of Cerebrovascular and neurodegenerative diseases, Tianjin dementia institute, Tianjin, Tianjin, China; ^2^ Tianjin Huanhu Hospital, Tianjin Key Laboratory of Cerebrovascular and neurodegenerative diseases, Tianjin dementia institute, Tianjin, Tianjin, China; ^3^ Beijing Friendship Hospital, Capital Medical University, Beijing, Beijing, China; ^4^ Department of Neurology, the Second Affiliated Hospital of Guangzhou Medical University, Institute of Neuroscience, the Second Affiliated Hospital of Guangzhou Medical University, Guangzhou, Guangdong, China; ^5^ West China Hospital of Sichuan University, Chengdu, Sichuan, China; ^6^ Shanghai Mental Health Center, Shanghai Jiaotong University School of Medicine, Shanghai, Shanghai, China; ^7^ Henan Provincial People's Hospital, Zhengzhou University People's Hospital, Zhengzhou, Henan, China; ^8^ Fujian Medical University Union Hospital, Fujian Key Laboratory of Molecular Neurology and Institute of Neuroscience, Fujian Medical University, Fuzhou, Fujian, China; ^9^ Clinical College of Neurology, Neurosurgery and Neurorehabilitation, Tianjin Medical University, Tianjin, Tianjin, China

## Abstract

**Background:**

Caregivers' decisions have a significant impact on the progression of Alzheimer's disease (AD). However, research exploring the benefits of disease‐modifying therapy (DMT) from the perspective of caregivers is limited. This study conducted a questionnaire survey among informal caregivers in families using lecanemab in China, aiming to explore their attitudes towards the diagnostic methods of AD and the application of lecanemab.

**Method:**

This cross‐sectional survey included 345 informal caregivers of Lecanemab‐treated patients. A 36‐item questionnaire was utilized to gather baseline information from caregivers, as well as their comprehension of AD and treatment decisions, and feelings following Lecanemab administration. We collected online questionnaires from 37 tertiary hospitals across 31 provinces/autonomous regions/municipalities in China (2024/06/24‐2024/12/24). Multivariate analysis was conducted by ordered logistic regression.

**Result:**

The participants' average age was 50.84, with an annual household income of ¥390,000 ($53,196). The average duration of care provided by caregivers is 2.37 years. In the selection of diagnostic methods for AD, 91.3% of caregivers chose PET, 20% chose blood, and 17.7% chose cerebrospinal fluid. However, caregivers still had insufficient understanding of AD disease and anti‐amyloid protein therapy. Approximately 94.5% of caregivers reported that the use of Lecanemab did not exacerbate their caregiving burden. After using Lecanemab, 64.0% of the caregivers held the opinion that the drug had reached the anticipated therapeutic efficacy, and 86.3% of the caregivers manifested their willingness to continue its application. Among them, the groups with higher annual household income (*p* < 0.001) and where the caregivers are the patients' children (*p* = 0.040) were significantly more satisfied with the efficacy of Lecanemab (Table 1). Compared with younger caregivers (*p* = 0.038) and those with an annual income below ¥200,000 ($27,280) (*p* = 0.006), elderly caregivers and those with an annual income ranging from ¥400,000 to ¥590,000 ($54,560 ‐ $80,476) were more inclined to continue using Lecanemab (Table 2).

**Conclusion:**

Most caregivers hold a positive attitude towards Lecanemab, particularly those with higher household incomes and when the caregivers are the patients' children. Elderly caregivers and those with an annual income of ¥400,000 to ¥590,000 were more inclined to continue using Lecanemab.